# Competence-based catalog of learning objectives for the subject area of quality management in medical studies – position paper of the working group Quality Management in Education, Training and Continuing Education of the Society for Quality Management in Health Care (GQMG)

**DOI:** 10.3205/zma001624

**Published:** 2023-06-15

**Authors:** Michael Vogeser, Kirstin Börchers, Janina James, Julian Koch, Doris Kurscheid-Reich, Silke Kuske, Barbara Pietsch, Susanne Zillich

**Affiliations:** 1 Klinikum der Universität München, LMU München, Institut für Laboratoriumsmedizin, Munich, Germany; 2 Universität Duisburg-Essen, Fakultät für Wirtschaftswissenschaften, Lehrstuhl für Medizinmanagement, Essen, Germany; 3 Universitätsklinikum Essen, Stabsstelle Qualitätsmanagement und klinisches Risikomanagement, Essen, Germany; 4 Klinikum der Universität München, LMU München, Klinik und Poliklinik für Frauenheilkunde und Geburtshilfe, Munich, Germany; 5 Städtisches Klinikum Solingen, Zentrales Qualitätsmanagement, Solingen, Germany; 6 Fliedner Fachhochschule Düsseldorf, Düsseldorf, Germany; 7 Gemeinsamer Bundesausschuss (until 2020), Berlin, Germany; 8 Universitätsklinikum Erlangen, Urologische und Kinderurologische Klinik, Erlangen, Germany

**Keywords:** quality management, medical studies, catalog of learning objectives, competencies, teaching

## Abstract

**Background:**

Traditionally, direct medical competences are taught in medical studies, whereas leadership and quality management competences are hardly taught, although graduates are already confronted with management tasks at the beginning of their clinical work. With the upcoming amendment of the Medical Licensing Regulations, this topic area will probably be addressed and must be adequately taught by the faculties. The learning objectives in the area of quality management listed in the current working version of the German National Catalogue of Learning Objectives in Medicine (NKLM) 2.0 have so far been formulated in rather general terms and need to be concretized.

**Aim:**

To develop a competence-based learning objectives catalog for the topic area of quality management in medical studies as a structured framework recommendation for the design of faculty teaching-learning programs and as a suggestion for further development of the NKLM.

**Methods:**

The competence-based learning objectives catalog was developed by an eight-member working group “Quality Management in Education, Training and Continuing Education” of the Gesellschaft für Qualitätsmanagement in der Gesundheitsversorgung e.V. (GQMG) within the framework of a critical synthesis of central publications. The members of the project group have many years of project experience in quality management in health care as well as in university didactics.

**Results:**

Six basic competence goals as well as 10 specific competence goals could be formulated and consented upon. These are each flanked by a list of essential basic concepts and examples. These focus on quality improvements, including patient safety and treatment success against the background of a physician leadership role in an interprofessional context.

**Discussion:**

A competency-based set of learning objectives has been compiled that encompasses the necessary concepts and basic knowledge of quality management required for those entering the profession to understand and actively participate in quality management after completing medical school. To the authors' knowledge, no comparable learning objectives catalog is currently available for medical studies, even internationally.

## Zusammenfassung

**Hintergrund:**

Traditionell werden im Medizinstudium überwiegend direkte medizinische Handlungskompetenzen vermittelt, Führungs- und Qualitätsmanagementkompetenzen hingegen kaum, obgleich Absolvierende bereits zu Beginn ihrer klinischen Tätigkeit mit Managementaufgaben konfrontiert sind. Mit der anstehenden Novellierung der Ärztlichen Approbationsordnung wird dieser Themenbereich voraussichtlich adressiert und ist von den Fakultäten entsprechend adäquat zu lehren. Die in der aktuellen Arbeitsfassung des Nationalen Lernzielkatalogs Medizin (NKLM) 2.0 aufgeführten Lernziele im Bereich des Qualitätsmanagements sind bisher eher allgemein formuliert und bedürfen einer Konkretisierung.

**Ziel:**

Die Entwicklung eines kompetenzbasierten Lernzielkatalogs für den Themenbereich Qualitätsmanagement im Medizinstudium als strukturierte Rahmenempfehlung für die Gestaltung fakultärer Lehr-Lernangebote und als Anregung für eine Weiterentwicklung des NKLM.

**Methoden:**

Der kompetenzbasierte Lernzielkatalog wurde durch eine achtköpfige Arbeitsgruppe „Qualitätsmanagement in der Aus-, Fort- und Weiterbildung“ der Gesellschaft für Qualitätsmanagement in der Gesundheitsversorgung e.V. (GQMG) im Rahmen einer kritischen Synthese zentraler Publikationen erarbeitet. Die Mitglieder der Projektgruppe besitzen langjährige Projekterfahrung im Qualitätsmanagement im Gesundheitsweisen sowie in der Hochschuldidaktik.

**Ergebnisse:**

Es konnten sechs grundsätzliche Kompetenzziele sowie 10 spezifische Kompetenzziele formuliert und konsentiert werden. Diese werden jeweils von einer Liste von wesentlichen Grundbegriffen und Beispielen flankiert. Diese fokussieren auf Qualitätsverbesserungen, einschließlich Patientensicherheit und Behandlungserfolg vor dem Hintergrund einer ärztlichen Führungsrolle im interprofessionellen Kontext.

**Diskussion:**

Es wurde ein kompetenzbasierter Lernzielkatalog zusammengestellt, der die notwendigen Konzepte und Grundkenntnisse des Qualitätsmanagements umfasst, die für Berufseinsteigende nach abgeschlossenem Medizinstudium erforderlich sind, um Qualitätsmanagement zu verstehen und aktiv daran partizipieren zu können. Nach Kenntnisstand der Autoren ist derzeit auch international kein vergleichbarer Lernzielkatalog für das Medizinstudium verfügbar.

## Introduction

Traditionally, medical studies prepare students for a specialist career. The focus is on teaching direct competencies in curative care, and to a lesser extent competencies in rehabilitation and prevention. However, doctors starting their careers are also confronted from the very beginning with leadership and management tasks – to which quality management clearly belongs – in sometimes complex interprofessional constellations. These tasks, which are not directly medical, increase in complexity and scope for most physicians throughout the course of their professional life [1]. In the currently valid licensing regulations for physicians in Germany, the topic of quality management is not specifically addressed. It can therefore be assumed that a large proportion of doctors starting their careers are currently experiencing a deficit in their training with regard to quality management, although this cannot currently be substantiated with scientifically collected data.

It is very likely that in the upcoming amendment of the medical licensing regulations, the subject area of leadership and management will be addressed in the category of higher-level competence. Accordingly, it is to be expected that all medical faculties in Germany will have to develop teaching programs that provide students with competencies in this area. In the context of the expected amended licensing regulations is the National Competence-Based Learning Objectives Catalog for Medicine (NKLM), which has been available as a working version in version 2.0 since mid-2021 [https://nklm.de/zend/menu]. In basic terms, the NKLM defines binding specifications for faculty teaching. A derived learning objectives catalog of the Institute for Medical and Pharmaceutical Examination Questions (IMPP) forms the basis for state examinations [2]. As of January 2023, the NKLM and IMPP catalog are essentially identical in text. In these present versions of the NKLM and IMPP catalogs of learning objectives, the area of leadership and management is mapped; among these, the two matching subchapters VII 5.5 address “models and methods of quality management”. The following competence objectives are formulated: *“The graduates knows models and methods of quality management and applies them. They have knowledge of quality assurance measures in patient care and their areas of application. They can explain the essential legal requirements of quality management (including quality assurance), name the relevant institutions for quality assurance in medicine, describe the necessity of structured measures of training and compulsory instruction. They know terms, concepts and tools of quality management and implement them. They can explain the importance of quality management in preparing for internal and external incidents, name and consider written organizational, work and treatment procedures, describe complaint management, name those involved in it and deal adequately with a specific complaint, critically reflect on relevant quality management documentation, actively participate in structured team meetings, explain the principles of clinical risk management.” *In the opinion of the GQMG-AG Quality Management in Education, Training and Continuing Education, these competence goals are relevant; however, they need to be supplemented and concretized in order to reflect at least the legally mandated minimum requirements for an institution's internal quality management and to be able to serve as a practice-oriented framework for the development of teaching offers of the faculties. In accordance with this assessment, the AG 2021 initiated a process for the development of a competence-based learning objective catalog for quality management for medical students as a first step. The aim was to develop a competence-based catalog of learning objectives for the subject area of quality management in medical studies as a structured guide for the design of faculty teaching and learning opportunities and as a stimulus for further development of the National Catalog of Learning Objectives in Medicine. In particular, the concepts and terminology of quality management anchored in Social Code V §§ 135 ff and in the binding quality management guideline of the Federal Joint Committee [3] should be taken into account.

## Methods

The Gesellschaft für Qualitätsmanagement im Gesundheitswesen e.V. (GQMG) is the German national medical-scientific professional society for the field of quality management and a corporate member of the Arbeitsgemeinschaft medizinisch-wissenschaftlicher Fachgesellschaften (AWMF). The board of the GQMG commissioned the GQMG working group Quality Management in Education, Training and Continuing Education to develop a learning objectives catalog for medical studies. This assignment was worked on by the eight-member working group beginning September 2021. All eight members of the interprofessional project group have many years of project experience in quality management in health care; this includes teaching activities at universities in the field of quality management, consulting in clinical risk management and general quality management, clinical medical activity, and activity and/or training as clinical quality management officers.

An extensive literature review, synthesis, and communicative validation of the results were conducted. For the synthesis of central competence-based learning objectives, the legally binding documents and subject-specific publications on quality management in health care were first used as a basis and evaluated in a critical synopsis: Social Code V (§§ 135 ff); Patients' Rights Act, Quality Management Guideline of the Joint Federal Committee [3]; Course Book on Medical Quality Management of the German Medical Association [4]; the German standard DIN EN 15224 Quality Management Systems for Health Care [5] as well as the catalog Terms and Concepts of Quality Management of the German Society for Medical Informatics, Biometry and Epidemiology e. V. (GMDS) and the GQMG [6]. A first list of elements, which were assessed as fundamental by the AG, was then compared with available relevant textbooks [7], [8] or further available literature [9], [10]. Furthermore, the search terms “quality management” in combination with “medical students”, “medical education”, “medical curriculum”, “medical/clinical learning objectives” were used to search PubMed and Google for further relevant publications under consideration of professional literature.

This means that the relevant core literature was taken into account, which reflects the current status of quality management, is anchored in law and consensual in the professional societies of quality management, and reflects the state of science on the subject. In particular, the corresponding guideline of the Joint Federal Committee [3] is guiding with regard to “internal quality management in facilities” according to the German Social Code V. In addition, terms that are widely used in medical quality management and that have proven useful in the teaching experience of the AG members for conveying and, if necessary, deepening the teaching content were named for explanation. The work also included experience gained in the implementation of an elective “introduction to quality management in medicine” since 2019 at the Medical Faculty of LMU Munich.

Based on the final included literature and the experience-based findings, appropriate operationalized competency-based learning objectives were formulated. Basic competencies and specific sub-competency goals were developed and synthesized. Here, a formulation of the competencies was based on the Higher Education Qualification Framework of the Conference of Ministers of Culture of 2017 and refers in essence to Bloom’s learning goal taxonomy. For the majority of the learning objectives, the competence level “knowledge of action and reasoning” is applied, corresponding to level 2 according to NKLM terminology (explaining facts and contexts, placing them in the clinical-scientific context, and evaluating them in a data-based manner) [https://nklm.de/zend/menu]. For a limited number of objectives, action competence under guidance was targeted (corresponding to 3 a). Competency levels are appropriately categorized in the catalog by verbs according to Bloom.

In developing it, an effort was made to ensure a pragmatic balance between minimum requirements and scope.

An initial draft of the learning objectives catalog created in this way was communicatively validated, further developed, and finally consented in several structured videoconferences and on the basis of circulated comment lists in the AG. This was followed by an evaluation and commentary of the working document by the GQMG board, resulting in the submitted publication version.

## Results

By consensus of the working group, a competence-based learning objectives catalog with six basic competence objectives and 10 specific competence objectives of physician quality management could be developed.

The six basic competence goals relate to (1) organizational measures to improve and maintain the success of treatment and patient safety. The (2) physician quality management is seen as a central leadership task, which (3) takes into account the interprofessional contexts of quality management in (medical) health care and the associated responsibilities. The (4) application of working methods (and tools) of quality management as well as for achieving goals in a medical institution is in focus as well as (5) the further development of quality management in a medical institution. Against this background, a corresponding (6) attitude, responsibility and reflection, combined with a respectful, positive image of human beings is to be achieved.

The basic competence objectives form a comprehensive framework which is further concretized by the specific competence objectives and can be shaped in detail by means of exemplary basic concepts as well as examples (cf. attachment 1 ). The specific competence objectives cover the basic concepts of quality management, areas of application of quality management, institutions, regulations (legal, non-legal) and standards, process management, document management, evidence-based decision support, competence management and communication, error management as well as clinical risk management and quality improvement. Accordingly, the tools to be taught range from quality management tools directly related to patients (such as learning and reporting systems), to ward-level processes (such as structured team meetings), to higher-level quality assurance processes at the institutional level, or to health policy quality concerns. Thematic points of contact and connection with several other subjects according to the medical licensing regulations should be recorded, e.g. medical informatics, hygiene, forensic medicine.

Beyond the sources mentioned in the methodology section of this manuscript, our internet-based research did not yield any previously published documents or catalogs that could have been considered for inclusion in the present learning objectives catalog. 

The details of the proposed learning objectives catalog on the topic area of quality management and the corresponding literature sources can be found in attachment 1 . 

## Discussion

The present document provides a comprehensive and well-founded catalog of basic and specific competence objectives in the subject area of quality management for medical students. The GQMG recommends this document as a basis and guideline for the curricular design of teaching in medical quality management by the medical faculties. Essentially, it addresses in a coherent form the requirements for internal quality management and inter-institutional quality assurance measures [3] that are legally anchored in Germany. The GQMG catalog is consistent with the learning objectives formulated in the currently published version of the NKLM on models and methods of quality management, but substantively concretizes them in the process. To the authors’ knowledge and according to their research, no comparable catalog is available internationally.

The proposed catalog covers the basic knowledge of quality management for physicians; it is not the intention of the document to cover the topic of quality management in medicine completely and exhaustively for all professional groups. Nevertheless, the interprofessional context is made explicit, as this corresponds to the reality of care. Quality management is taught as a medical management task, whereby a functioning quality management necessarily requires the expert participation of all those active in the interprofessional context of medical care. In this context, however, students should also be able to recognize dimensions that are specifically medical in nature. In particular, the systematic medical evaluation of clinical treatments and outcomes should be mentioned here, but ultimately also the overall medical responsibility for a respective medical institution.

The application of general principles and procedures of quality management to the field of patient care should enable a goal-oriented, efficient health care system to achieve the best possible care with the given resources. The primary intention is to show students a practice-oriented and low-threshold approach to the profoundly positive, patient- and staff-oriented principles of modern quality management philosophy in medicine.

Quality management in medicine must always take into account the special situation of medical practice. Physicians bear a high ethical and social responsibility. They are not first and foremost obligated to the system, but primarily to the people entrusted to their care within the framework of the given possibilities. This sometimes presents physicians with extraordinary challenges, for which they should be prepared with appropriate expertise.

Globally, there are a number of medical teaching curricula that more or less exclusively address the topic of patient safety without comprehensively depicting comprehensive and proactive quality management for error prevention; in particular, the curriculum proposed by the WHO [11] in the context of the WHO Global Action Plan for Patient Safety 2021-2030 [12], the patient safety learning objectives catalog formulated by the GMA [13], and the catalog available from the Patient Safety Action Alliance [14] should be mentioned here. However, an acceptable level of patient safety is hardly conceivable without mastered processes at the facility level and without effective quality management. Accordingly, specific patient safety curricula should be considered as complementary to the comprehensive scope of quality management in healthcare.

In addition, for medical students who have acquired basic knowledge and competencies in quality management on the basis of the GQMG catalog, the ability to continue their education after medical licensure is ensured – especially with regard to the curriculum of the German Medical Association for the additional title of medical quality management [4].

The catalog of learning objectives presented here does not yet include recommendations for implementation at the level of teaching-learning opportunities; the development of practical teaching concepts in this topic is to be regarded as the next step, which will be included in the further work of the GQMG-AG. Furthermore, the authors are of the opinion that the development and implementation of teaching-learning formats for medical quality management should be accompanied by research activities in order to be able to objectify the initial situation and successes [15]. This will also be the subject of further work by the WG.

The continuous improvement process is an essential basic motive of quality management, which of course also has to apply to the GQMG catalog – as well as to the NKLM. Accordingly, the GQMG invites all interested parties to participate in the joint further development of the catalog and to accompany the further activities of the WG.

## Conclusion

The GQMG learning objectives catalog can serve as a basis to support medical faculties in the development of teaching-learning offers in this subject area with a well-structured topic grid of medical quality management in medical studies. It can also serve as a stimulus for the further development of the NKLM/IMPP learning objectives catalog.

## Competing interests

The authors declare that they have no competing interests. 

## Supplementary Material

Competency-based learning objectives catalog Quality management in healthcare
