# Supplementary material for: Competence-based catalog of learning objectives for the subject area of quality management in medical studies – position paper of the working group Quality Management in Education, Training and Continuing Education of the Society for Quality Management in Health Care (GQMG)
Source: GMS J Med Educ. 2023 Jun 15;40(4):Doc42. doi: 10.3205/zma001624 (PMC10407586; doi:10.3205/zma001624)
Supplement: Competency-based learning objectives catalog Quality management in healthcare [file JME-40-42-s-001.pdf]

## Attachment 1: Competency-based learning objectives catalog Quality management in healthcare

| | | |
|---|---|---|
| | **Basic competence goals**<br><br>a) The graduates explain quality management (QM) in health care as the totality of organizational measures in medical facilities that serve to improve or maintain the success of treatment and patient safety,<br><br>b) they identify QM as a central management task; they explain its importance for clinical patient care and the processes in medical facilities,<br><br>c) they highlight the interprofessional context of QM in health care and derive specifically medical aspects and responsibilities,<br><br>d) they apply selected essential working methods (and tools) of QM in a medical institution and name the respective goals,<br><br>e) they design their own contributions to the further development of QM in a medical facility,<br><br>f) they explain that QM is based on attitude, responsibility, reflection, respect and a positive image of humanity. | |
| **Chap.** | **Specific competence goals** | **Exemplary basic terms and examples** |
| **1.** | **Basic concepts of quality management**<br><br>1.1 The graduates explain essential basic terms of quality management and are able to apply them to medical facilities across all occupational groups<br><br>1.2 They explain the basic requirements for the management of medical facilities<br><br>1.3 They identify quality management as a prerequisite for a high-quality, efficient and safe practice of medicine | • Quality definition<br>• Quality dimensions: Structural quality, process quality, outcome quality, indication quality.<br>• Quality characteristics<br>• Management, management system, quality management, integrated management<br>• Quality management system<br>• Quality capability, quality objectives, quality steering<br>• Quality control, quality assurance<br>• Quality requirements in health care |

Attachment 1 to Vogeser M, Börchers K, James J, Koch J, Kurscheid-Reich D, Kuske S, Pietsch B, Zillich S. Competence-based catalog of learning objectives for the subject area of quality management in medical studies – position paper of the working group Quality Management in Education, Training and Continuing Education of the Society for Quality Management in Health Care (GQMG). GMS J Med Educ. 2023;40(4):Doc42. DOI: 10.3205/zma001624

| | | |
|---|---|---|
| **2.** | **Areas of application of quality management**<br><br>2.1 They explain the different levels at which quality management is implemented in medical facilities.<br><br>2.2 They name the typical tasks of a QM representative / QM coordinator.<br><br>2.3 They formulate the specific role and perspective of the physician with regard to QM in an interdisciplinary team (especially with regard to clinical outcome assessment and overall responsibility) | • QM measures directly related to patients (e.g., patient identification wristbands; team time-out procedure; WHO preoperative checklist).<br>• QM at ward or practice level (e.g., structured team meetings)<br>• QM at the level of clinics and institutions (e.g., QM and patient safety staff units)<br>• Tasks of higher-level, external institutions with regard to QM and quality assurance (see also Chapter 3) |
| **3.** | **Institutions, regulations (legal, non-legal) and standards**<br><br>3.1 They name the institutions relevant for quality assurance in medicine in Germany and their tasks<br><br>3.2 They name essential legal regulations as well as non-legal guidelines and standards and describe their essential contents | **Institutions, including:**<br>• Federal Ministry of Health (BMG), state ministries of health<br>• Joint Federal Committee (G-BA) with its scientific institutes "Institute for Quality and Efficiency in Health Care" (IQWiG) and "Institute for Quality Assurance and Transparency in Health Care" (IQTIG)<br>• German Medical Association (BÄK), State Medical Associations (LÄKn)<br>• Medical service of the health insurance (MDK)<br><br>**Regulations (laws, guidelines, rules, standards), including:**<br>• Social Code (SGB) V<br>• Guidelines and regulations of the G-BA<br>• Medical professional code of conduct<br>• Guidelines of the German Medical Association<br>• Patients' Rights Act<br>• Medical Device Operator Ordinance<br>• Transfusion Act<br>• Infection Protection Act<br><br>**Norms / standards, including:**<br>• DIN EN ISO 9001, DIN EN 15224:2016, KTQ, EFQM |

| | | |
|---|---|---|
| **4.** | **Process management**<br><br>4.1 They explain the definition, optimization, monitoring and control of processes in terms of process organization as a core element of quality management<br><br>4.2 They identify patient safety and successful implementation of quality improvement interventions as an essential quality feature of medical care<br><br>4.3 They explain essential terms, concepts and tools of process management and apply them to medical facilities | • Process types (core process, support process, management process; key processes)<br>• Process chains, process landscapes<br>• Process owners, process participants, process goal, process end, process key figures, process risks, process evaluation<br>• Interfaces<br>• Handovers (patients, documents, samples, devices, etc.)<br>• Change management |
| **5.** | **Document management**<br><br>5.1 They interpret the regulation of the handling of documented information as a core element of quality management<br><br>5.2 They apply the essential terms, concepts and tools of document management | • Documented information<br>• Document categories (standard documents, records; differentiation from patient-related documentation)<br>• Document types (esp. forms, checklists, notices, procedural instructions, work instructions, equipment books, medication plans, QM manual)<br>• Versioning<br>• Relational documentation, unambiguity and consistency<br>• Other applicable documents<br>• 4-eyes principle<br>• Document control |
| **6.** | **Evidence-based decision support**<br><br>6.1 They explain the principles of evidence-based decision making, including structured information gathering and assessment as the basis for patient care based on scientific evidence | • Information and knowledge management<br>• Evidence based medicine<br>• Guidelines, directives |

| | | |
|---|---|---|
| **7.** | **Competence management and communication**<br><br>7.1 They explain the definition of structure, affiliations, instruction relationships, responsibilities and competences in terms of an organizational structure as a core element of quality management<br><br>7.2 They present the essential terms, concepts and tools of competence management and can explain them for medical institutions | • Responsibility, function, delegation<br>• Organization charts<br>• Job description, job specification<br>• Further education, training, induction<br>• Training planning, authorization<br>• Supervision concepts<br>• Competence matrix<br>• Competence review<br>• Delegation fault, takeover fault<br>• Device responsibility and device instruction in accordance with the German Medical Devices Act<br>• Appointment of representatives (e.g., safety officers, data protection officers)<br>• Structured team meetings<br>• Physician reservation and formal qualification requirements, specialist standard |
| **8.** | **Error management**<br><br>8.1 They highlight the structured handling of errors as a core element of quality management<br><br>8.2 They explain the essential terms, concepts and tools of error management with regard to medical facilities | • Error term<br>• Error chains<br>• Active and latent errors<br>• Factors promoting errors in complex care situations<br>• Error log<br>• Error analysis (influencing factors, causes, extent)<br>• Corrective measures<br>• Preventive measures<br>• Effectiveness testing of measures<br>• Complaint management<br>• Non-conformity with regard to normative requirements<br>• Culture of failure |

Attachment 1 to Vogeser M, Börchers K, James J, Koch J, Kurscheid-Reich D, Kuske S, Pietsch B, Zillich S. Competence-based catalog of learning objectives for the subject area of quality management in medical studies – position paper of the working group Quality Management in Education, Training and Continuing Education of the Society for Quality Management in Health Care (GQMG). GMS J Med Educ. 2023;40(4):Doc42. DOI: 10.3205/zma001624

| | | |
|---|---|---|
| **9.** | **Clinical risk management**<br><br>9.1 They explain that the elements of quality management in health care are indispensable prerequisites for achieving an acceptable level of patient safety in medical facilities of any kind<br><br>9.2 They highlight that quality management of healthcare facilities includes clinical risk management as a continuous process (risk analysis, risk assessment, risk control, risk monitoring)<br><br>9.3 They explain the essential terms, concepts and tools of risk and safety management<br><br>9.4 They identify typical deficiencies in the clinical context that pose a risk to safety and communicate them appropriately to the situation<br><br>9.5 They reflect personal attitude and attitudes with regard to risk minimization<br><br>9.6 They derive clinical safety management as an interaction of different disciplines such as hygiene, pharmacology, anesthesiology, medical informatics, etc. | • Safety and risk terms<br>• Error, undesirable event, serious undesirable event, damage, fault<br>• Risk analysis and risk catalog<br>• „Never events“<br>• Risk assessment, risk control, risk monitoring<br>• Risk minimization, risk audit<br>• Communication-related risks (e.g., language barriers, abbreviations)<br>• Organizational culpability and system factors favoring errors<br>• Crisis management; continuity management, emergency plans<br>• Mass casualty incidents or infected patients<br>• Special measures to ensure patient safety (e.g., patient identification bracelets, fall prevention)<br>• Checklists for repetitive standard procedures<br>• CIRS, risk reporting systems / reporting and learning systems<br>• Risks of hierarchies and personal conflicts, "human factor<br>• Second victim concept<br>• Employee safety |

| | | |
|---|---|---|
| **10.** | **Quality improvement**<br><br>10.1 They interpret the continuous development and improvement of structures, processes and outcomes in the context of patient care as a central objective of quality management for the entire institution<br><br>10.2 They explain and evaluate essential concepts and tools of continuous improvement processes | • Structured survey of patients, employees and referring physicians<br>• Internal and external audits<br>• Benchmarking<br>• Complaint management<br>• Quality targets<br>• Analysis of opportunities and development possibilities<br>• Certification, accreditation<br>• Continuous improvement process (CIP)<br>• PDCA cycle<br>• Action plans<br>• Management review<br>• Project Management |
